# Kernel score statistic for dependent data

**DOI:** 10.1186/1753-6561-8-S1-S41

**Published:** 2014-06-17

**Authors:** Dörthe Malzahn, Stefanie Friedrichs, Albert Rosenberger, Heike Bickeböller

**Affiliations:** 1 Department of Genetic Epidemiology, University Medical Center, Georg-August University Göttingen, Humboldtallee 32, 37073 Göttingen, Germany

## Abstract

The kernel score statistic is a global covariance component test over a set of genetic markers. It provides a flexible modeling framework and does not collapse marker information. We generalize the kernel score statistic to allow for familial dependencies and to adjust for random confounder effects. With this extension, we adjust our analysis of real and simulated baseline systolic blood pressure for polygenic familial background. We find that the kernel score test gains appreciably in power through the use of sequencing compared to tag-single-nucleotide polymorphisms for very rare single nucleotide polymorphisms with <1% minor allele frequency.

## Background

Lately, much interest has been focused on global multilocus procedures that test for overall association of sets of markers. This is appealing if the loci carry rare variants or are thought to belong to a specific gene set, such as a pathway or genes with similar functions. The kernel score statistic is a specific type of such a global covariate-adjusted multilocus association test. Depending on context, it has received different names, such as SKAT (sequence kernel association test) [1]. Kernel methods provide flexible semiparametric regression models of multimarker genetic influence on expected trait outcome and can conveniently be implemented within the linear mixed-model formalism [2,3]. The kernel expresses genetic correlation between subjects. It allows us to test a whole model class by defining a prior probability in function space in a bayesian manner [4]. In contrast, standard regression models are limited to just 1 *a priori *stated submodel of such a class.

Until very recently, kernel methods were only applied to independent data (see Ref. [5] for a review). An extension to include familial dependence was made for trait prediction [6], but not for testing genetic influence. This changed with independent Genetic Analysis Workshop 18 (GAW18) contributions by ourselves and others (Huang et al [7], Chen et al [8], Dufresne et al [9], and Schifano et al [10]). Huang et al [7] applied the kernel method to trios. The other 4 contributions (including ours) use a similar kernel extension to families with different study aims. We generalize the kernel score statistic to adjust for dependency in metric trait data. We apply it to real and simulated GAW18 baseline systolic blood pressure (SBP) and contrast its performance on the sequenced panel in comparison to tag-single-nucleotide polymorphisms (SNPs) of a genome-wide association study (GWAS).

## Methods

### Data

GAW18 provided blood pressure trait data from Mexican Americans participating in the San Antonio Family Heart Study or the San Antonio Family Diabetes/Gallbladder Study, and in the Type 2 Diabetes Genetic Exploration by Next-generation sequencing in Ethnic Samples (T2D-GENES) Project 2 study [11]. The sample is enriched for type 2 diabetes and for rare variants. Complete dense SNP allele dosage data were provided for 959 subjects from 20 large pedigrees with 22 to 86 members. Data are based on genome-wide tag-SNP genotyping of all subjects, whole-genome sequencing of 464 selected subjects, and imputation of all missing dosages for the remaining SNPs and subjects, exploiting kinship within pedigrees [11]. We considered only subjects with known baseline SBP, sex, and age, who were not on blood pressure medication: 706 subjects with real SBP, excluding the first listed twin of monozygotic twin pairs and 740 to 781 subjects with simulated SBP (subject numbers vary for 200 simulated study replicates as a result of inclusion criteria). We tested candidate genes on chromosome 3 only (real SBP: 5 genes selected based on previous association reports [12], simulated SBP: gene *MAP4*). From group discussions, we knew that *MAP4 *associates with simulated SBP and that simulated trait Q1 mimics the segregation of genes within families, but otherwise represents the genetic null hypothesis.

### Kernel score statistic with adjustment for random confounders

We analysed baseline SBP, which has a right-skewed distribution toward large extremes. This was amended by a priori rank-normalization (Blom transformation [13]) of SBP to a standard normally distributed target variable Y_i _= F^−1^((r_i_-3/8)/(n_obs_+1/4)). F^−1 ^denotes the standard normal distribution quantile; n_obs _the number of observed SBP values; and r_i _their respective rank. Individual traits Y_i _for i = 1, ..., n subjects were modeled to depend on fixed effects **b **of covariates **X**_*i*_ (intercept, age, sex, age × sex interaction), on random effects **c **for shared familial polygenic background, and on a semiparametric model h(**G**) of a genetic marker set **G**.

(1) $${\text{Y}}_{\text{i}}\sim {\text{X}}_{\text{i}}{\text{b}}^{\text{T}}+{\text{Z}}_{\text{i}}{\text{c}}^{\text{T}}+\text{h(}\text{G}\text{)}$$

Vectors **X**_i _and **Z**_i _represent the subject rows of respective design matrices **X**, **Z **for fixed covariate effects and random family effects. The semiparametric model h(**G**) can be written as **h**=**Ka**^T ^with n × n dimensional kernel matrix **K **and multivariate normally distributed random effects estimates **a**~*N*(0,τ**K**) [2]. The kernel score statistic tests for a genetic covariance component τ with null hypothesis H_0_: τ = 0. Most conveniently, it can be computed based on restricted maximum likelihood parameter estimates of the genetic null model

(2) $$\text{Y}={\text{Xb}}_{\text{o}}^{\text{T}}+{\text{Zc}}_{\text{o}}^{\text{T}}+\text{e},$$

which estimates only fixed covariate effects **b_o_**, random pedigree effects **c_o_**, polygenic component variance s^2^_fam _and residual variance s^2^, **e**~*N*(0,s ^2^**I**). Extending the kernel score statistic to adjust for familial dependencies, we obtain test statistic

(3) $$\text{Q}={\left(\text{Y}-{\text{Xb}}_{\text{o}}^{\text{T}}\right)}^{\text{T}}{\text{V}}_{\text{o}}^{-1}{\text{KV}}_{\text{o}}^{-1}\left(\text{Y}-{\text{Xb}}_{\text{o}}^{\text{T}}\right)/\text{2}$$

with matrix **V**_o _= s^2^**I**+s^2^_fam_**ZZ**^T^. With fixed effects estimate **b_o_=**(**X**^T^**V**_o_^−**1**^**X**)^−**1**^**X**^T^**V**_o_^−**1**^**Y**, we rewrite test statistic (3) as quadratic form Q=**R**^T^**MR **of standard normally distributed residuals **R**= **P**_o_^1/2^**Y **with matrix **M=**(**P**_o_^1/2^**K P**_o_^1/2^)/2 and null projection matrix **P_o_=V_o_^−1 ^- V_o_^−1^X(X^T^V_o_^−1^X)^−1^X^T^V_o_^−1^**. The *p *values for test statistic (3) can be calculated by the Davies exact method [14] with the R package CompQuadForm from sample estimates Q and all eigenvalues of matrix **M**. We adjusted for polygenic familial background based on the kinship coefficient matrix Φ_kin_=**ZZ**^T ^using R-packages kinship2 and coxme with R-function lmekin for genetic null model (2). We employed a linear kernel on allele dosages. This allows for additive genetic models on tested markers. The kernel matrix entry **K**_ij_=**g**_i_^T^**g**_j _for 2 subjects i, j is the scalar product of their subject-specific vectors **g**_i_, **g**_j _of allele dosages for N_SNP _SNP marker. SNPs were included for testing if their Hardy-Weinberg equilibrium test *p *values ≥10^−5 ^(rounding imputed dosages for this purpose only). We contrasted test performance for the same subjects on 2 SNP panels, sequence (allele dosage data) versus GWAS (allele dosage data reduced to intersection with GWAS SNPs).

## Results

Permutation testing demonstrated overall test validity: 50,000 permutations of subject-genotype assignment (permuting across families) yielded type I error 0.049 ± 0.0019, 0.00046 ± 0.00019 at significance level 0.05, 0.0005 for simulated SBP on all sequenced rare *MAP4 *SNPs. Simulated trait Q1 was used to test the quality of polygenic adjustment. We analyzed Q1 analogously to SBP on gene *MAP4 *and obtained *p *values ≤0.05 with rate 0.02 (all SNPs, and SNPs with minor allele frequency (MAF) >5%), rate 0.05 (for MAF 1% to 5%), and 0.06 (for MAF <1%). This is within 95% tolerance limits (0.02, 0.08) for true value 0.05 on 200 replicates.

**Figure 1:**
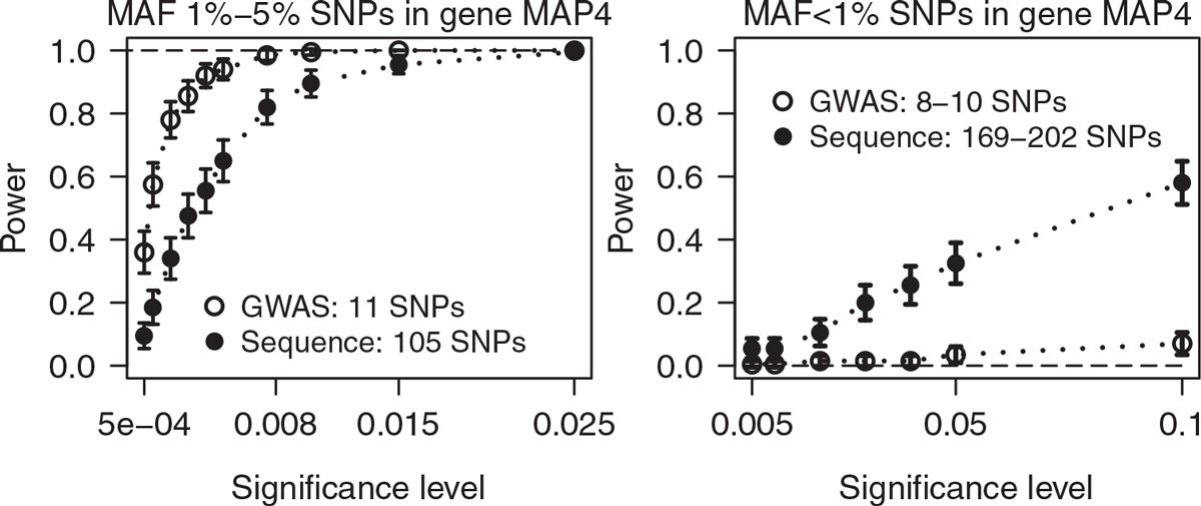
**Simulated SBP associates with gene *MAP4*** . Power estimates of the kernel score statistic with 95% confidence limits over 200 simulated study replicates as function of the significance level. The association between rank-normalized simulated systolic blood pressure and *MAP4 *was tested, adjusting for age, sex, age × sex interaction, and familial polygenic background. Polymorphic SNP numbers vary between study replicates for very rare SNPs with MAF <1% (range in legend, *right panel*), but not for MAF 1% to 5% (*left panel*).

For simulated SBP, associated gene *MAP4 *was detected with 100%, 62.5%, and 21.5% power at significance levels 0.05, 10^−4^, and 10^−8^ on 200 study replicates when subjecting the *MAP4 *sequence to the global test. Power diminished by only 0% to 2.5% when using the GWAS panel instead, or only MAF >5% SNPs. Figure 1 displays power to detect *MAP4 *association based on MAF ≤5% SNPs. We varied the significance level to compare test performance on the sequence (filled circles) with the considerably sparser GWAS panel (open circles). Sequenced data are powerful and outperform GWAS data particularly for very rare SNPs (MAF <1%) where GWAS had no power (Figure 1, *right*). GWAS outperformed sequence data on SNPs with MAF 1% to 5% (Figure 1, *left*) with just 11 tag-SNPs compared to 105 sequenced SNPs.

Table 1 displays association of real SBP with the 5 selected candidate genes on chromosome 3. *P *values do not withstand Bonferroni correction. Note, however, the difference between sequence (*p *= 0.150) and GWAS (*p *= 0.031) for *MECOM *SNPs with MAF 1% to 5%. For *AGTR1*, sequence data tend to reduce *p *values compared to GWAS, as well as for *SLC4A7 *on rare variants. We partitioned the large *MECOM, ULK4 *sequences based on number of SNPs, as we expect that power improves by changing from a situation where we test over many more SNPs compared to number of subjects (full gene) to a situation with fewer SNPs than subjects (gene parts).

**Table 1:** Real SBP and candidate genes on chromosome 3

Gene	SNP panel	Kernel score statistic (linear kernel on allele dosage data)							
		
		All SNPs		MAF >5%		MAF 1% to 5%		MAF <1%	
		N_SNP_	*p *Value	N_SNP_	*p *Value	N_SNP_	*p *Value	N_SNP_	*p *Value
*MECOM*	GWAS	212	0.840	176	0.873	33	**0.031**	3	0.333
	Sequence	2530	0.826	1222	0.864	621	0.150	687	0.289
	Sequence	- 4 consecutive windows							
		633	0.976	364	0.977	93	0.482	176	0.890
		633	0.557	229	0.709	198	**0.0071**	206	0.197
		632	0.251	340	0.252	161	0.371	131	0.137
		632	0.919	289	0.933	169	0.467	174	0.216

*ULK4*	GWAS	188	0.770	146	0.783	40	0.258	2	0.430
	Sequence	3333	0.510	1569	0.518	1007	0.302	757	0.459
	Sequence	- 5 consecutive windows							
		667	0.661	314	0.648	177	0.499	176	0.301
		667	0.791	373	0.812	125	0.171	169	0.409
		667	0.738	296	0.813	208	0.108	163	0.446
		666	0.132	261	0.124	256	0.364	149	0.432
		666	0.141	325	0.132	241	0.540	100	0.830

*PPARG*	GWAS	148	0.693	90	0.664	32	0.880	26	0.998
	Sequence	583	0.841	269	0.803	121	0.989	193	0.990

*AGTR1*	GWAS	39	0.277	27	0.296	11	0.097	1	0.482
	Sequence	497	0.081	174	0.091	172	0.100	151	0.223

*SLC4A7*	GWAS	27	0.688	13	0.789	10	0.134	4	0.208
	Sequence	375	0.776	149	0.864	82	0.099	144	0.158

Kernel score test over N_SNP _SNP marker for rank-normalized systolic blood pressure, adjusted for age, sex, age × sex interaction, and familial background (*p *≤0.05 in bold).

## Conclusions

The kernel score statistic is a global covariance component test. *P *values for genes are dominated by their common SNPs (see Table 1: all SNPs vs. MAF >5%). Thus, common and rare SNP sets should be tested separately. The kernel method offers a simple and flexible way to perform multimarker analysis and genetic interaction analyses by means of the kernel choice. It improves power compared to single-marker tests by exploiting SNP correlation [10], and if jointly tested SNP sets tag multiple independent associated loci. The test statistic by Liu et al and Tzeng et al [2,3] had 17% power at the 0.01 level for independent subjects to find *MAP4 *association with simulated SBP in 200 replicates. Our extension to families increased this to 98.5% power, used 84% more subjects with twice as many polymorphic SNPs (sequence), without type I error inflation. Ignoring familial dependency would inflate type I error to 16%, 6% at nominal 0.05, 0.01 levels (applying the independent subjects test statistic [2,3] to families for *MAP4 *on polygenic trait Q1). We found that sequencing can improve test power appreciably, particularly for MAF <1% variants (Figure 1, *right*). For more frequent SNPs, the comparison was not decisive and tag-SNPs proved to be strong competitors. With sequencing, even a single, large gene may provide more SNP variants than available study subjects. The kernel score test remains valid because of the Bayesian nature of the underlying nonparametric genetic model [4]. However, one might expect power loss. The kernel score statistic may be susceptible to random confounders. For example, simplistic adjustment of familial and common cultural effects by a familial random intercepts model (instead of the polygenic model) yields valid permutation test results, but shows up as inflated type I errors on simulated polygenic trait Q1 (*p *≤0.05 on 10% of 200 study replicates for all *MAP4 *SNPs, inflation increases with decreasing MAF). This, in turn, would yield similar but overly optimistic estimates for Figure 1 and Table 1. With such a liberal test, *AGTR1 p *values in Table 1 would all drop below 0.05 for all sequence SNP sets. We would like to emphasize that other random confounder effects, such as different trait variability between study subgroups, can be adjusted in exact analogy to the familial polygenic background adjustment.

## Competing interests

The authors declare that they have no competing interests.

## Authors' contributions

DM designed the study concept, developed the statistical method, conducted data extraction, all statistical analyses and data interpretation, and drafted the manuscript. SF and AR provided the SNP mapping to positions (NCBI build 37) and genes. HB contributed throughout with discussions. All authors read and approved the final manuscript.

## References

[B1] Wu MC Lee S Cai T Li Y Boehnke M Lin X Rare-variant association testing for sequencing data with the sequence kernel association test Am J Hum Genet 2011 89 82 93 10.1016/j.ajhg.2011.05.029 21737059 PMC3135811

[B2] Liu D Lin X Ghosh G Semiparametric regression of multidimensional genetic pathway data: least-squares kernel machines and linear mixed models Biometrics 2007 63 1079 1088 10.1111/j.1541-0420.2007.00799.x 18078480 PMC2665800

[B3] Tzeng J-Y Zhang D Haplotype-based association analysis via variance-components score test Am J Hum Genet 2007 81 927 938 10.1086/521558 17924336 PMC2265651

[B4] Rasmussen CE Williams CK Gaussian Processes for Machine Learning 2006 Cambridge, MA, MIT Press

[B5] Schaid DJ Genomic similarity and kernel methods I: advancements by building on mathematical and statistical foundations Hum Hered 2010 70 109 131 10.1159/000312641 20610906 PMC7077093

[B6] Gianola D van Kaam JB Reproducing kernel Hilbert spaces regression models for genomic assisted prediction of quantitative traits Genetics 2008 178 2289 2303 10.1534/genetics.107.084285 18430950 PMC2323816

[B7] Huang J Chen Y Swartz M Ionita-Laza I Comparing the power of family-based association test for sequence data with applications in the GAW18 simulated data BMC Proc 2014 8 suppl 1 S27 10.1186/1753-6561-8-S1-S27 PMC4143708 25519316

[B8] Chen H Choi SH Hong J Lu C Milton JN Allard C Lacey SM Lin H Dupuis J Rare genetic variant analysis on blood pressure in related samples BMC Proc 2014 8 suppl 1 S35 10.1186/1753-6561-8-S1-S35 PMC4143757 25519320

[B9] Dufresne L Oualkacha K Forgetta V Greenwood CMT Pathway analysis for genetic association studies: To do, or not to do, that is the question BMC Proc 2014 8 suppl 1 S103 10.1186/1753-6561-8-S1-S103 PMC4144468 25519357

[B10] Schifano ED Epstein MP Bielak LF Jhun MA Kardia SL Peyser P Lin X SNP set association analysis for familial data Genet Epidemiol 2012 36 797 810 22968922 10.1002/gepi.21676 PMC3683469

[B11] Almasy L Dyer TD Peralta JM Jun G Fuchsberger C Almeida MA Kent JW Jr Fowler S Duggirala R Blangero J Data for Genetic Analysis Workshop 18: Human whole genome sequence, blood pressure, and simulated phenotypes in extended pedigrees BMC Proc 2014 8 suppl 1 S2 10.1186/1753-6561-8-S1-S2 PMC4145406 25519314

[B12] SNPedia August 2012 http://www.snpedia.com

[B13] Blom G Statistical Estimates and Transformed Beta Variables 1958 New York, John Wiley & Sons

[B14] Davies R Algorithm as 155: the distribution of a linear combination of chi-2 random variables J R Stat Soc Ser C 1980 29 323 333

